# Counseling at work place: A proactive human resource initiative

**DOI:** 10.4103/0019-5278.40807

**Published:** 2008-04

**Authors:** Smita Navare

**Affiliations:** Trustee Drishti Human Resource Centre Contact : 09323496704 E-mail: naren.smta@gmail.com

Peter Drucker one of the greatest management thinkers of our time says “In a few hundred years, when the history of our time is written from a long term perspective, it is likely that the most important event the historians will see is not technology, not the Internet, nor e commerce. It is the unprecedented change in the human condition.

For the first time – literally – substantial and rapidly growing number of people have choices. For the first time they will have to manage themselves, and society is totally unprepared for it”.

The following statements reinforce the above statement:

“With the recent restructuring and the faster cycle of business, My staff is finding work pressure difficult to cope with. I am finding it hard to coach them to cope with the transition.” Concerned Line Manager

“I have become very anxious lately due to my heavy workload and family responsibilities. I start taking medicine to calm myself, but it doesn't seem to help.” Worried Employee

“Findings from our employee survey show a worrying trend of loss of work-life balance. We need to do something before this starts harmpering Productivity and staff well-being.” Human Resources Director

Counseling is definitely one service that can help people learn to manage themselves.

Different people since the beginning of mankind e.g. Parents, teachers, friends, elders, etc have used counseling in some way or the other. It was to the family doctor that people went most frequently.

Today of course it is a very specialized service and a profession in itself. Pressure at work place and at home, lack of support system such as elders and the mismatch of expectations are the reasons for disharmony between couples & there is alienation.

Tata Consultancy Services (TCS) has set up a network “Maitree” in 2005 to counsel its 30,000 employees. Under the initiative, 90% of TCS offices organize family get-togethers and activities such as ball dancing and yoga classes and theatre workshop, helping employees working long hours keep healthy.

“At Wipro, to reduce employee stress after long working hours, HR initiated “Mitr”, an in house counseling service, in 2003, the set up trains employees in counseling to help out colleagues in distress,” said a senior HR manager with Wipro Technologies”

Recent tragic death of young couple made headlines in the print media were not so fortunate, Only if they had called the 24x7 helpline Infosys set up two years ago the out come would not have been tragic.

Professional counselors who can stimulate personal growth in others; offer help in addressing many situations that cause emotional stress, including, but not limited to:

- Anxiety, depression, and other mental and emotional problems and disorders
- Family and relationship issues
- Substance abuse and other addictions
- Sexual abuse and domestic violence
- Absenteeism
- Career change and job stress
- Social and emotional difficulties related to disability and illness
- Adopting to life transitions
- The death of a loved one
- Appropriate referrals after assessment.

Good indicators of when you should seek counseling are when you're having difficulties at work, your ability to concentrate is diminished or when your level of pain becomes uncomfortable.

**Dimensions that can be added to workplace counseling:**

- Managers could be trained in some basic counseling skills.
- Some growth and development workshops can be conducted in emotional Intelligence, Transactional Analysis, Marriage enrichment etc can also be conducted by the counselor.

A counselor in a work place can work with designated personnel as a thinking partner, a revealing mirror, and a pacesetter among others.

A counselor can help in leveraging core capacities of employees.

Can help create a culture for greater synergy in organizational learning and development.

Can help employees increase their self-awareness regarding their thinking patterns and behavioral tendencies so as to make them more effective as an individual and in turn effective in their job also.

Managers know that many problems at work often stem from an employee's personal life rather than just the work situation.

What we do from 9 to 5 at job is directly related to what we do between 5 to 9 outside the work place.

Counseling is an effective and preventive people management strategy for organizations to help employees better managing stress, personal issues or work related problems.

**Benefits to the organization:**

- Decrease costs related to turnover, burnouts, absenteeism & accident-related disability.
- Improvement in employee performance & therefore increase in productivity.
- Counselor can play the role of a business partner to manage behavioral Problems brought about by organizational changes.
- Unfortunately our educational system does not equip us with living skills.

A counselor can train people in managing themselves and thus enhancing Personal growth.

## COUNSELORS EXPERTISE

The person practicing counseling could be a Psychiatrist, a psychologist,

A psychotherapist or a social worker or any other person trained in counseling.

The person of the counselor, the maturity, knowledge and experience are all significant for counseling to be effective.

Above all the counselor should vouch complete confidentiality.

Counselors need to give assurance of absolute confidentiality.

Quarterly statistical reports to Management that quantifies utilization rates and share trends of issues for which consultation is sought while adhering to the principle of confidentiality.

Health Services need to also incorporate Personal counseling as part of their services. It requires a respectable place for a counselor where an individual can experience privacy to express himself / herself.

Managing self or Life Style is very much in the purview of Health and thus a counselor can add value to the services provided for “Wellness”.

There are a few good counselors practicing in India but efforts needs to be done to build capacity in this profession. The counseling service at work place shall bring back work life balance and serve as developmental model rather than problem solving model.

